# Diet-induced Pigmented Purpuric Dermatosis Confirmed with a Rechallenge Response

**DOI:** 10.7759/cureus.5273

**Published:** 2019-07-29

**Authors:** Wendy Li, Matthew Reedy, Ahmed K Alomari, Sahand Rahnama-Moghadam

**Affiliations:** 1 Medicine, Indiana University School of Medicine, Indianapolis, USA; 2 Dermatology, Indiana University School of Medicine, Indianapolis, USA; 3 Dermatology, Pathology, Indiana University School of Medicine, Indianapolis, USA

**Keywords:** diet-induced, food, pigmented purpuric dermatosis, rechallenge

## Abstract

The pigmented purpuric dermatoses (PPDs) are a group of chronic cutaneous eruptions characterized by non-blanching and non-palpable purpuric lesions. Their etiology is not completely understood, although dietary exposures have been implicated in a few case reports. We describe a recurring case of diet-induced PPD in a 73-year-old Caucasian male following the ingestion of tomato-based products on two separate occasions, one year apart. On physical examination, he demonstrated numerous 1-2 mm red/brown, non-blanching, petechial macules scattered on the bilateral anterior lower legs, thighs, trunk, arms, hands, and feet with facial sparing. Histopathologic examination revealed the classic perivascular lymphocytic infiltrate with red blood cell extravasation seen in PPD. Mirroring his first episode, the patient saw a complete resolution of his rashes with careful avoidance of tomato-based products and required no other interventions. This represents a rare case of diet-induced PPD confirmed with a rechallenge response and suggests that acute or recurrent cases of PPD may be a result of a hypersensitivity reaction.

## Introduction

The pigmented purpuric dermatoses (PPDs) are a group of chronic cutaneous eruptions that are histologically similar. They are traditionally categorized into five clinical subtypes, although numerous other variants exist: progressive PPD (Schamberg's disease), lichen aureus, eczematid-like purpura of Doucas-Kapetanakis, purpura annularis telangiectodes (Majocchi disease), and Gougerot-Blum purpura. These clinical subtypes represent morphologic variations of the same underlying disorder, which has been hypothesized to be related to increased capillary fragility and/or an idiopathic cell-mediated inflammatory response [1-3].

PPD has been associated with venous hypertension, exercise, infections, alcohol, contact allergens, drugs (i.e., acetaminophen, aspirin, diuretics), and systemic diseases (i.e., hyperlipidemia, diabetes, hepatitis) [1]. However, very few cases of diet-induced PPD have been described in the literature to date. We present a rare case of diet-induced PPD diagnosed following re-exposure to a known dietary trigger with rapid resolution after discontinuation.

## Case presentation

**Figure 1:**
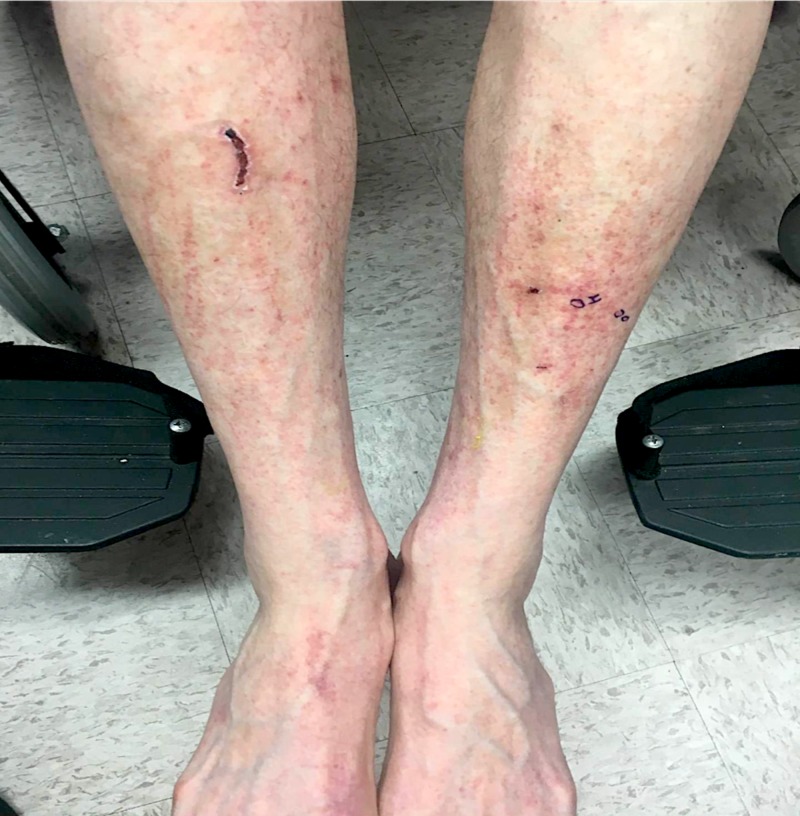
Clinical manifestation on anterior lower legs

**Figure 2:**
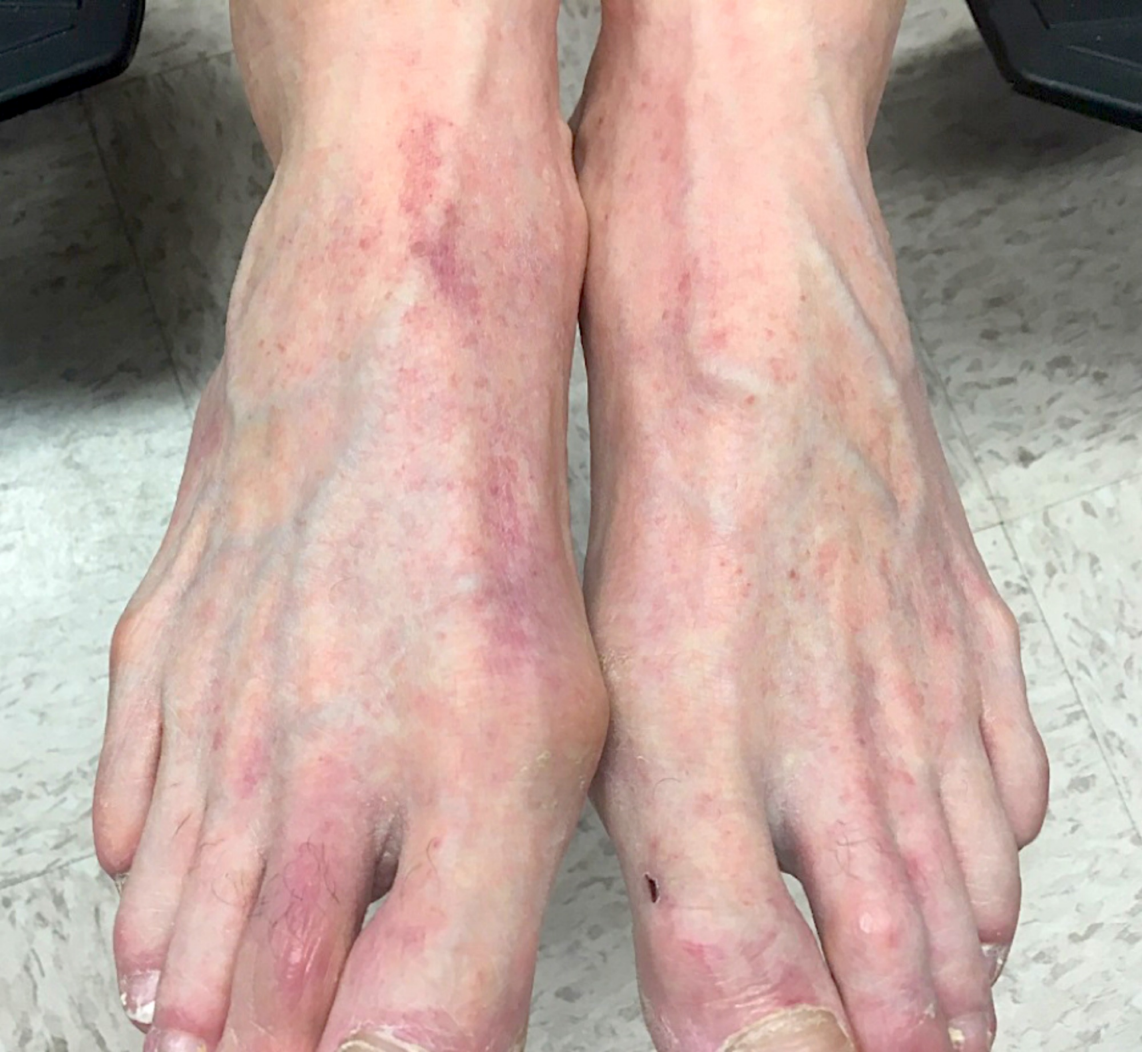
Clinical manifestation on dorsal feet

**Figure 3:**
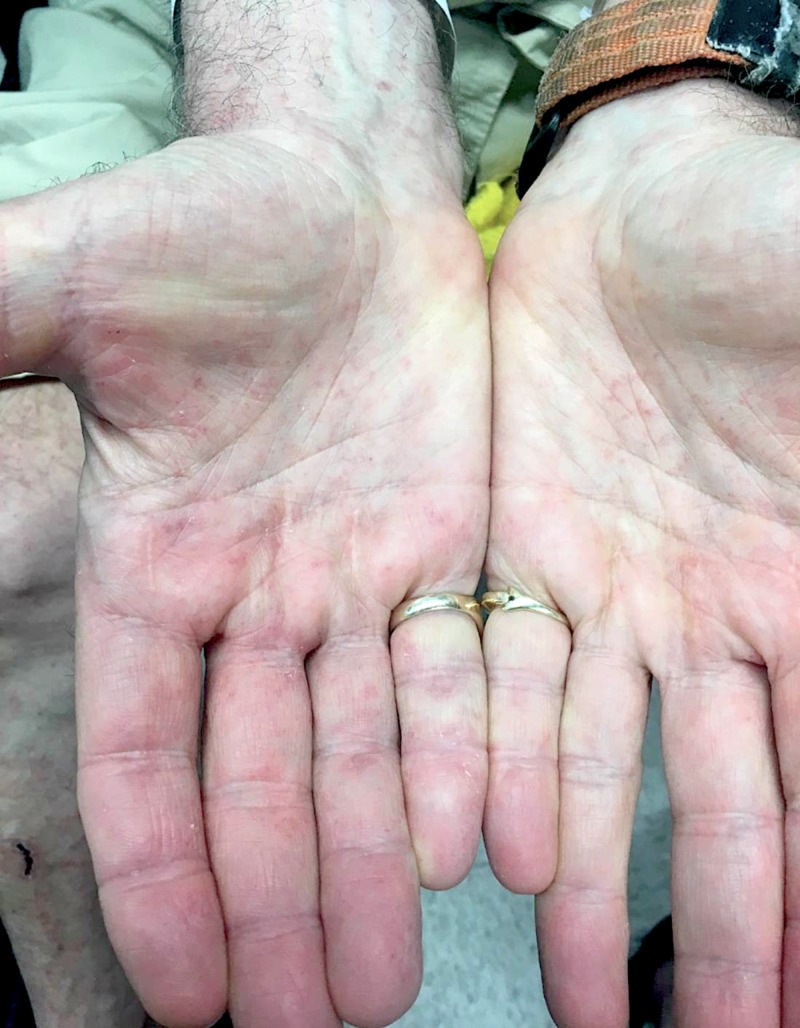
Clinical manifestation on palmar hands

A 73-year-old Caucasian man presented with a five-day history of a mildly pruritic rash that appeared the next morning after eating spaghetti with tomato sauce. He recalled a very similar rash one year prior shortly after eating fresh tomatoes. At that time, he avoided tomatoes and tomato-based products and noticed rapid clearing of his skin. On this instance, he wanted to rechallenge himself with tomato sauce, but developed a more widespread rash compared to his first episode. Review of systems was negative. He denied new medications, supplements, or illnesses prior to the eruption. On examination, the patient demonstrated numerous 1-2 mm red/brown, non-blanching, petechial macules scattered on the bilateral anterior lower legs, thighs, trunk, arms, hands, and feet with facial sparing (Figures 1, 2, 3).

**Figure 4:**
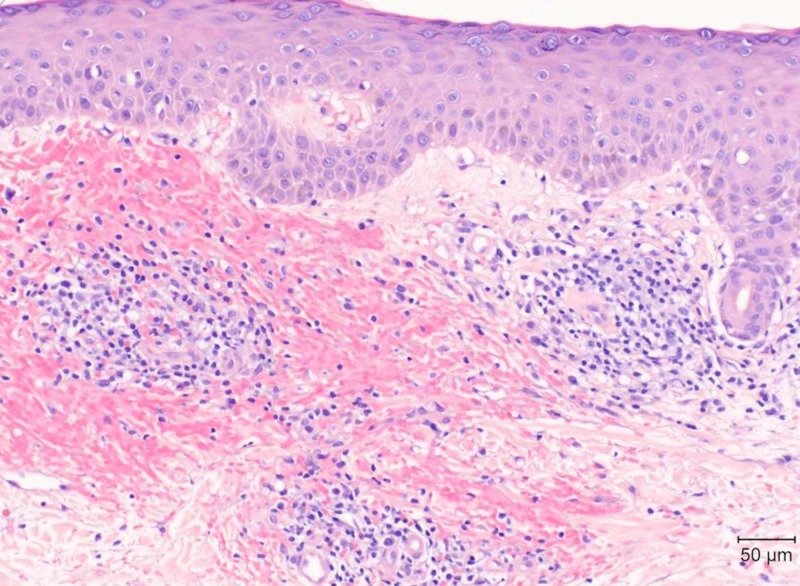
Hematoxylin and eosin-stained section of punch biopsy sample at 200x magnification Findings: superficial perivascular lymphocytic infiltrate with red blood cell extravasation and mild interface damage

Punch biopsy of the left anterior leg was subsequently performed. Histopathology revealed superficial perivascular lymphocytic infiltrate with red blood cell extravasation, mild interface damage, and no evidence of leukocytoclastic vasculitis (Figure 4).

On direct immunofluorescence, there was weak and nonspecific granular basement membrane immunoreactant deposition with no perivascular localization. Laboratory tests including complete blood count, complete metabolic panel, and urinalysis were within normal limits. After the diagnosis was made, the patient was advised to avoid tomatoes. His lesions were only minimally symptomatic, so no treatment was deemed necessary. When the patient returned two weeks later for suture removal, the rash had completely resolved.

## Discussion

In PPD, both the chronic leakage of red blood cells through capillaries and hemosiderin deposition contribute to the non-blanching, petechial macules as seen in our patient. Differential diagnoses include, but are not limited to, cutaneous leukocytoclastic vasculitis, drug hypersensitivity reactions, benign hyperglobulinemic purpura, stasis dermatitis, and mycosis fungoides [2-4]. No laboratory abnormalities are associated with PPD. The classic histopathologic findings of PPD include a perivascular lymphocytic infiltrate, erythrocyte extravasation, and hemosiderin deposition. Immunofluorescence studies are usually negative [2].

Few cases of diet-induced PPD exist in the literature, and even fewer with a documented rechallenge response. Therefore, it is important to emphasize that the development of PPD in our patient: 1) followed a probable temporal sequence after a dietary trigger was ingested, 2) resolved on withdrawal of the dietary trigger, and 3) reappeared on rechallenge of the dietary trigger. In addition, evaluation using the Naranjo scale yields a score of 10 (Table 1).

**Table 1:** Naranjo Adverse Drug Reaction Probability Scale Scoring: less than or equal to 0 (doubtful); 1 to 4 (possible); 5-8 (probable); greater than or equal to 9 (definite)

Naranjo Adverse Drug Reaction Probability Scale				
Question	Yes	No	Do Not Know	Score
Are there previous conclusive reports on this reaction?	+1	0	0	1
Did the adverse event appear after the suspected drug was administered?	+2	-1	0	2
Did the adverse event improve when the drug was discontinued or a specific antagonist was administered?	+1	0	0	1
Did the adverse event reappear when the drug was readministered?	+2	-1	0	2
Are there alternative causes that could on their own have caused the reaction?	-1	+2	0	2
Did the reaction reappear when a placebo was given?	-1	+1	0	0
Was the drug detected in blood or other fluids in concentrations known to be toxic?	+1	0	0	0
Was the reaction more severe when the dose was increased or less severe when the dose was decreased?	+1	0	0	0
Did the patient have a similar reaction to the same or similar drugs in any previous exposure?	+1	0	0	1
Was the adverse event confirmed by any objective evidence?	+1	0	0	1
Total Score				10

Any score greater than or equal to 9 indicates a “definite” causal relationship between the dietary trigger and the development of PPD [5]. 

Furthermore, a careful review of the literature highlights possible distinctions between classic PPD and diet-induced PPD. Widespread skin involvement, as seen in our patient, is commonly reported in diet-induced PPD [6-8]. This is unusual because classic PPD is usually limited to the lower extremities. Interestingly, all identified cases of diet-induced PPD report rapid improvement or complete resolution upon discontinuation of the dietary trigger without the need for pharmacologic intervention (Table 2).

**Table 2:** Case Reports of Diet-induced Pigmented Purpuric Dermatosis

Case	Trigger	Intervention	Outcome	Rechallenge	Ref.
1.	Coca-cola, apple-cherry fruit spritzer	Discontinuation	Improvement	Yes, positive response	[6]
2.	Energy drink (caffeine, taurine, glucuronolactone)	Discontinuation	Complete resolution	No	[7]
3.	Dietary supplement (Iodine)	Discontinuation	Complete resolution	No	[8]
4.	Dietary supplement (creatine, hydroxymethylbutyrate)	Discontinuation	Complete resolution	No	[9]
5.	Dietary supplement (parselenium E)	Discontinuation	Complete resolution	No	[10]
6.	Dietary supplement (Thiamine)	Discontinuation	Improvement	Yes, positive response	[11]

In fact, our patient experienced complete resolution in just two weeks. This is fairly extraordinary because classic PPD usually follows a chronic course and is rather resistant to treatment [1-2]. One natural implication of our case is that acute or recurrent cases of PPD may be a result of a hypersensitivity reaction. Investigation of possible dietary triggers may help others to further elucidate the pathogenesis of this condition and also help individual patients avoid triggers.

## Conclusions

This represents a rare case of diet‐induced PPD in which a causal relationship between a dietary trigger and the development of PPD is established. In classic PPD, spontaneous resolution is very rare and pharmacologic treatments often yield inconsistent or ineffective results. However, discontinuation of the dietary trigger is the definitive treatment in our patient as well as in previous cases of diet-induced PPD. These outcomes imply that acute or recurrent cases of PPD may be the result of a hypersensitivity reaction. There is also evidence to suggest that diet-induced PPD may differ from classic PPD not only in the extent of skin involvement but also in prognosis. 
